# 

**Published:** 1888-10-13

**Authors:** 


### CONTENTS.

**The Rating of Charities** - 17

**The Phonograph in Hospital** - 18

**Hospital Finance and Economy.**—By a MEDICAL SUPERINTENDENT - 19

**Within the Wards.**—Knock-knees.—A Long Case - 20

**The Doctor's Pulpit.**—The Seven Ages of Man—II. The Schoolboy - 21

**Round About the Asylums, XIV.**—By a MEDICAL SUPERINTENDENT - 22

**The National Pension Fund** - 23

**Notes and News** - 24

**Everybody's Page** - 26

**Annotations.**—A Defender of Women.—Fallen Women.—Malthus and the Vicar - 27

**Voyages in Vacation, III.**—Is Sea-Sickness a Myth? - 28

**False Impressions.** By CAROLINE FOTHERGILL, Author of "An Enthusiast," "A Voice in the Wilderness," etc. - 29

**The Shaftesbury Memorial** - 31

**Turning Over New Leaves.**—"Sketches of Hospital Life" 31

**Amusements and Relaxation** - 32

**EXTRA SUPPLEMENT.—THE NURSING MIRROR.**

En Passant.—French Sisters of Charity.—A Nurses' Home for Sunderland.—Handbooks on Nursing.—An Indian Incident.—Yellow Fever in Florida.—Proper Training.—The Registration of Midwives.—Religion and Nursing - v
Notes from Australia.—The Alfred Hospital - vi
Appointment —Notice - vi
Everybody's Opinion.—The Whitechapel Murder.—Nurses who are Roman Catholics.—The Question of Courage.—Teachers of Culture - vii
Vacancies.—Examination Questions.—Notes and Queries - vii

